# A Child-Mother

**Published:** 1873-11

**Authors:** A. R. Kilpatrick

**Affiliations:** Navasota, Texas


A Child-Mother.—*A. R. Kilpatrick, M.D., Navasota, Texas.*—I was recently informed that a copper-colored negro girl, not twelve years old, of this (Grimes) county, gave birth, in the first week of last August, to a fully developed female child. There was some difficulty and delay in the parturition, but there was no mechanical assistance rendered. The mother and child have done well up to this time; lactation natural.

The mother and grandfather of the girl say she was about eleven and half years old when the child was born, and their testimony is corroborated by white persons who have known them several years. She has two older sisters, the eldest of whom would be considered young to have a child. The child was not weighed at birth, but the accoucheur says it was about seven and a half or eight pounds.

The German Schools of Medicine.—The number of students in the Vienna Medical Faculty diminishes sadly from year to year. Since Oppolzer's and Skoda's death, the diminution is very marked. Where one could with difficulty get within ear-shot in days gone by, on account of the number attending, the wards now, it is stated, are almost empty. In Prague and Gratz, however, the attendance is usually good.

